# The Interface of Additive Manufactured Tungsten–Diamond Composites

**DOI:** 10.3390/ma18112574

**Published:** 2025-05-30

**Authors:** Xuehao Gao, Dongxu Cheng, Zhe Sun, Yihe Huang, Wentai Ouyang, Cunxiao Lan, Zhaoqing Li, Lin Li

**Affiliations:** 1 Ningbo Institute of Materials Technology and Engineering, Chinese Academy of Sciences, Ningbo 315201, China; gaoxuehao@nimte.ac.cn (X.G.);; 2 Laser Processing Research Centre, Department of Mechanical, Aerospace and Civil Engineering, The University of Manchester, Manchester M13 9PL, UK; 3 National Key Laboratory for Remanufacturing, Army Academy of Armored Forces, Beijing 100072, China; 4 School of Science, Hubei University of Technology, Wuhan 430068, China

**Keywords:** laser powder bed fusion (L-PBF), tungsten, diamond, metal matrix composites, interface bonding

## Abstract

Tungsten–diamond metal matrix composites (MMCs) fabricated via L-PBF show potential for applications in nuclear facility shielding, heat sinks, precision cutting/grinding tools, and aerospace hot-end components. In this paper, tungsten (W), diamond (D), and diamond with Ni coating (D-Ni) powders are used to fabricate W+D and W+(D-Ni) composites by L-PBF technology. The results show that at the interface of the W+D sample, the W powder melts while the D powder remains in a solid state during L-PBF processing, and W and C elements gradually diffuse into each other. Due to the high cooling rate of L-PBF processing, the C phase forms a diamond-like carbon (DLC) phase with an amorphous structure, and the W phase becomes a supersaturated solid solution of the C element. At the interface of the W+(D-Ni) sample, the diffusion capacity of Ni and W elements in the solid state is weaker than in the molten state. C and W elements diffuse into the Ni melt, forming a rich Ni area of the DLC phase, while Ni and W elements diffuse into the solid D powder, forming a lean Ni area of the DLC phase. In the rich Ni area of the DLC phase, Ni segregation leads to the precipitation of nanocrystals (several hundred nanometers), whereas in the lean Ni area of the DLC phase, the diffusion capacity of Ni and W elements in the solid D powder is limited, resulting in nanocrystalline sizes of only about tens of nanometers. During W dendrite growth, the addition of the Ni coating and the expelling of the C phenomenon leads to W grain refinement at the interface, which reduces the number and length of cracks in the W+(D-Ni) sample. This paper contributes to the theoretical development and engineering applications of tungsten–diamond MMCs fabricated by L-PBF.

## 1. Introduction

Tungsten (W) has the advantages of high melting point, density, hardness, and fracture toughness, as well as excellent resistance to neutron radiation and chemical corrosion [1,2,3], which has been extensively implemented in many fields, such as nuclear power [1] and precision machining [2,3]. Diamond (D) possesses ultra-high hardness (70–150 GPa), excellent thermal conductivity (3.2 W/(cm·K)), and stable chemical properties [4]. Tungsten–diamond metal matrix composites (MMCs) combine the advantages of tungsten alloys and diamond, offering the potential for extreme temperature resistance, high thermal conductivity, high hardness, and good fracture toughness, making them highly suitable for nuclear facility protection and precision machining [1,2,3,4,5].

Because of the extreme hardness of tungsten alloys and diamonds and the heterogeneity of tungsten–diamond MMCs, conventional casting/forging methods are unsuitable for their fabrication, and machining remains challenging [6,7,8,9]. Currently, the predominant fabrication methods for tungsten-diamond metal matrix composites (MMCs) [8,9,10,11] include high-temperature and high-pressure sintering, vacuum hot-pressure sintering, spark plasma sintering, pressureless melt infiltration, and gas-pressure-assisted melt infiltration. However, these fabrication methods typically rely on forming molds, and the shape structure, dimensional size, and forming accuracy of tungsten-diamond MMCs are often constrained by traditional fabrication techniques and mechanical machining.

Additive manufacturing can form complex components point by point and layer by layer, which has been a promising technology for preparing metal–matrix diamond MMCs [12,13,14,15,16], including binder jet, ultrasonic additive manufacturing, cold spraying, laser-directed energy deposition, and laser powder bed fusion. Compared to other additive manufacturing technologies, laser-powder bed fusion (L-PBF) exhibits higher forming accuracy and quality, garnering widespread attention in metal–matrix diamond MMCs [17,18,19,20,21,22,23,24]. Yingbo Peng et al. [18] investigated the thermo-mechanical evolution of high-entropy alloy-diamond composites fabricated via L-PBF. Lu Zhang et al. [19] employed L-PBF technology to fabricate high-precision MMC metamaterial TPMS structures, which demonstrate significant potential in energy conversion, heat management, and lightweight applications. Yakun Tao et al. [20] analyzed the effect of Cu coating on the bonding properties of diamond/Cu-Sn matrix composites. Yingbo Peng et al. [21] studied the balling behavior and diamond graphitization of metal–matrix diamond composites by L-PBF. Kai Li et al. [22] fabricated Mo-coated diamond particle-reinforced metal matrix composites using L-PBF and investigated their mechanical behavior. Ruochong Wang et al. [24] analyzed the influence of diamond–powder physical properties and powder spreading parameters on powder bed quality and particle dynamics. However, tungsten-diamond MMCs prepared by L-PBF have rarely been studied.

In addition, during the L-PBF process of metal–matrix composites, diamond powder is prone to graphitization, and metal coatings are often used to prevent this phenomenon, such as Ti-Ni [18], Cu [19,20], and Mo [22] coatings. In addition, these metal coatings can improve the bonding force between the metal matrix and diamond powder, enhancing the comprehensive properties of metal matrix composites. However, tungsten-diamond MMCs with metal coatings have not been researched, and the effects of metal coatings on the element diffusion and microstructure formation at the interface between the metal matrix and diamond powder require urgent investigation.

In this paper, tungsten (W), diamond (D), and diamond with Ni coating (D-Ni) powders are used to fabricate W+D and W+(D-Ni) composites by L-PBF. The element diffusion behavior and microstructure evolution at the interface between W and carbon-phase in W+D and W+(D-Ni) composites are also studied. The interface research in this paper contributes to the theoretical development and engineering application of tungsten-diamond MMCs by L-PBF.

## 2. Experimental Materials and Methods

### 2.1. Material Preparation

**Figure 1:**
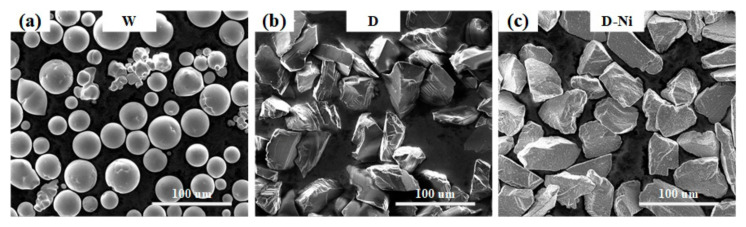
(**a**), (**b**), and (**c**) are SEM images of W, D, and D-Ni powders, respectively.

The sizes of the tungsten (W), diamond (D), and diamond with Ni coating (D-Ni) powders used in this study are listed in Table 1, and their morphologies are shown in Figure 1. The Ni coating was electroplated onto the D-Ni powder. The purities of the W powder, D powder, and Ni coating were above 99.9 wt%. The D-Ni powder showed a weight increase of 56% compared to the D powder. The 85 vol% W–15 vol% D powder and the 85 vol% W–15 vol% (D-Ni) powder were pre-mixed via a Y-type powder blender and are abbreviated as W+D and W+(D-Ni), respectively. The mixing time exceeded 30 min to ensure the uniformity of the powder mixtures. The L-PBF equipment used in this study was self-developed by our team. The L-PBF processing parameters for tthe W+D and W+(D-Ni) composite samples are as follows [25]: layer thickness 30 µm, laser power 400 W, hatch spacing 100 µm, and scanning speed 725 mm/s.

### 2.2. Material Characterization

The morphology, microstructure, and element distribution of the powders and sample surfaces were analyzed using scanning electron microscopy (SEM; Zeiss Sigma FEG, Oberkochen, Germany) equipped with energy-dispersive spectroscopy (EDS). The phase characteristics were examined by X-ray diffraction (XRD; D8 Advance, Bruker, Germany) with a scanning angle of 30–90° and a scanning speed of 4°/min. A Raman spectrometer (Renishaw InVia, New Mills, UK) with a 633-nm excitation laser was employed to detect the graphitization of diamonds in the samples. Transmission electron microscopy (TEM; Talos F200X, ThemoFisher, Waltham, MA, USA) equipped with energy-dispersive X-ray spectroscopy (EDX) was used to observe the microstructure and element distribution. Specimens for TEM observation were prepared using a focused ion beam (FIB; Helios G4 CX, Thermo scientific, Waltham, MA, USA).

## 3. Results

### 3.1. Morphology and Defects

**Figure 2:**
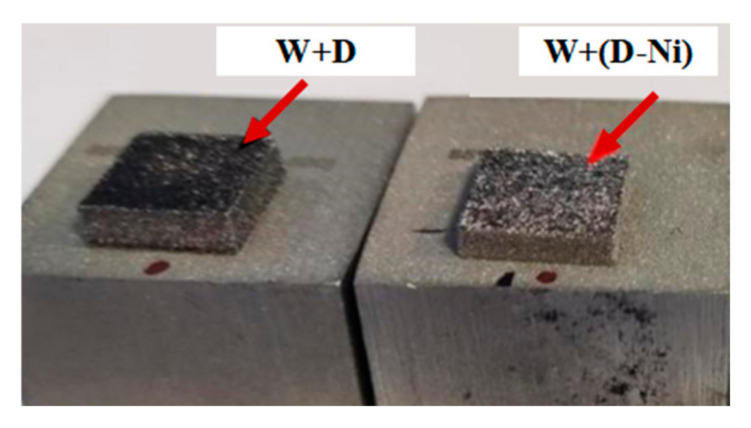
W+D and W+(D-Ni) composites from this study.

**Figure 3:**
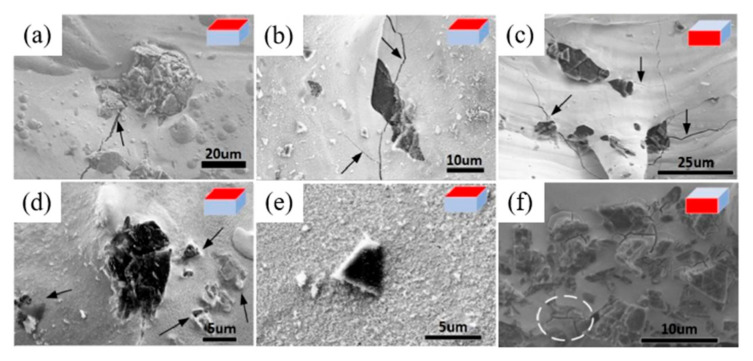
(**a**,**b**) show the top surface of the W+D sample; (**c**) shows the side surface of the W+D sample; (**d**,**e**) show the top surface of the W+(D-Ni) sample; (**f**) shows the side surface of the W+(D-Ni) sample. The cracks is marked by black arrows and white circle.

The appearances of the fabricated samples are shown in Figure 2 and Figure 3. The surface of the W+D sample appears black, while the W+(D-Ni) sample exhibits a more pronounced metallic color, as shown in Figure 2. The black surface of the W+D sample is attributed to diamond graphitization, whereas the Ni coating in the W+(D-Ni) sample inhibits this process, consistent with the XRD, TEM, and Raman results. Numerous microcracks are observed on both the top and side surfaces of the W+D sample, penetrating the W matrix and diamond particles, as indicated by the black arrows in Figure 3a–c. In contrast, fewer cracks are present around the diamonds in the W+(D-Ni) sample, as shown in Figure 3d–f, which is related to grain refinement of the W matrix at the interface (see detailed discussion in Section 4.2).

### 3.2. Phase Analysis of XRD

**Figure 4:**
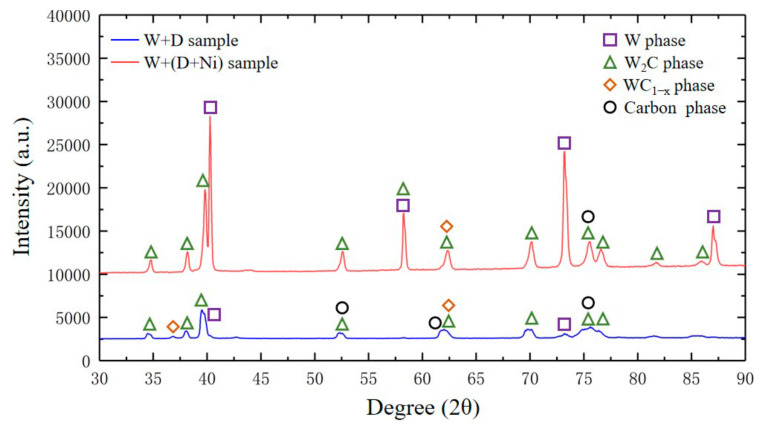
XRD results of W+D and W+(D-Ni) samples.

**Figure 5:**
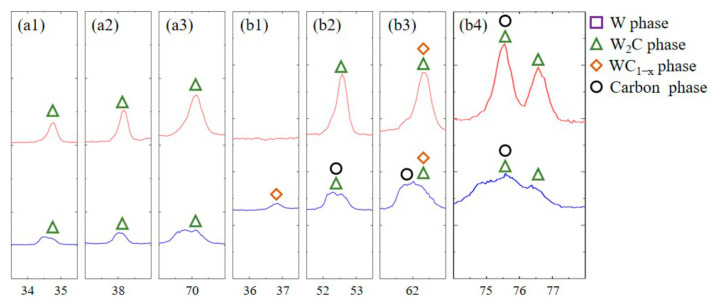
Enlarged view of the characteristic peaks from Figure 4. (**a1**–**a3**) are the contrast of W_2_C phase peaks, and (**b1**–**b4**) are the contrast of WC_1−x_ and carbon phase peaks.

According to the XRD results, the W+D and W+(D-Ni) samples primarily consist of W, W_2_C, WC_1−x_, and a carbon phase. The Ni coating suppresses W_2_C phase formation and carbon phase graphitization (Figure 4 and Figure 5). In the W+(D-Ni) sample, W is the dominant phase, followed by W_2_C as the secondary phase. In contrast, the W+D sample shows a lower intensity of W characteristic peaks and a reduced W phase volume fraction. The WC_1−x_ and carbon phase peaks are relatively weak, representing minor phases in both samples. Furthermore, the W+D sample exhibits lower peak heights for W_2_C, WC_1−x_, and the carbon phase compared to W+(D-Ni), along with diffuse peak patterns (Figure 5). These phenomena are attributed to the amorphization and graphitization of the carbon phase in the W+D sample [26,27,28], consistent with TEM and Raman results.

### 3.3. Phases Structure and Composition

**Figure 6:**
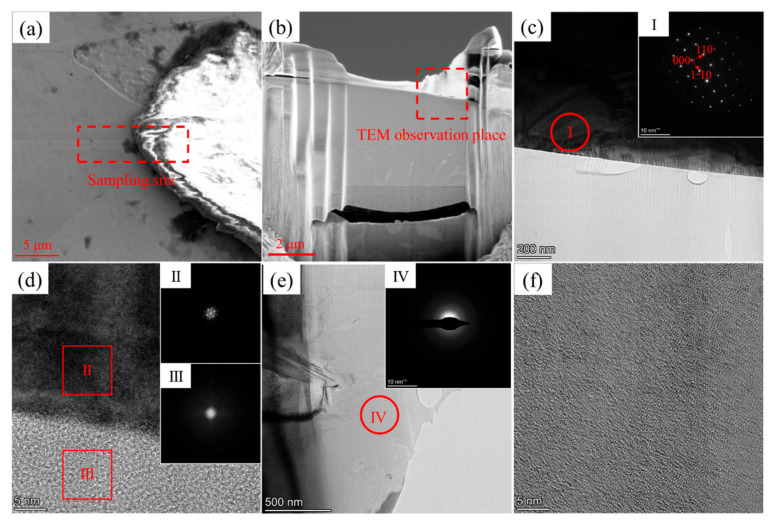
Phase structure of W and carbon phase interface in the W+D sample: (**a**) TEM sampling site; (**b**) TEM sample prepared by FIB; (**c**) bright-field TEM image of the interface—upper side is the W phase, lower side is the carbon phase, with the diffraction pattern of the W phase shown in image I; (**d**) HRTEM image near region I of (**c**). Regions II and III are FFT images of the W and carbon phases, respectively; (**e**) bright-field TEM image of the carbon phase, with the diffraction pattern of the carbon phase shown in image IV; (**f**) HRTEM image at region IV of (**e**).

**Figure 7:**
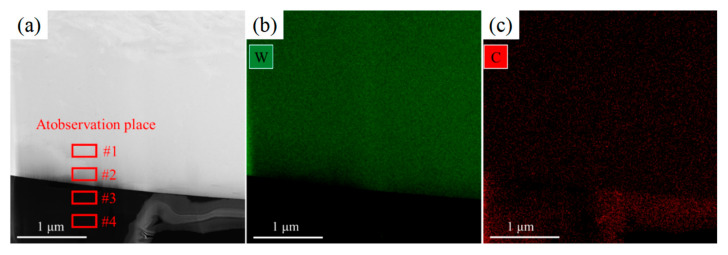
Composition distribution at the W and carbon phase interface in the W+D sample: (**a**) dark-field TEM image—upper side is the W phase, lower side is the carbon phase; (**b**) EDX mapping of W element; (**c**) EDX mapping of C element. Composition of Zone #1–#4 is shown in Table 2.

The phase structure at the W and carbon phase interface in the W+D sample is shown in Figure 6. The W phase exhibits a β-bcc structure, as confirmed by its diffraction pattern, as shown in Figure 6c,d. The carbon phase at the interface displays a halo diffraction pattern, as shown in Figure 6d,e, indicative of amorphization, while HRTEM images reveal a disordered carbon structure, as shown in Figure 6d–f, further corroborating this phenomenon. Based on previous studies [26,27,28], this amorphous carbon phase corresponds to diamond-like carbon (DLC). In the W+D sample, W and C elements undergo gradual interdiffusion (Figure 7 and Table 2). Within the W phase, the C content decreases from 11.62 at% at the interface (Zone #2) to 8.45 at% in the interior (Zone #1). Conversely, in the carbon phase, the W content decreases from 2.32 at% at the interface (Zone #3) to 1.54 at% in the interior (Zone #4).

**Figure 8:**
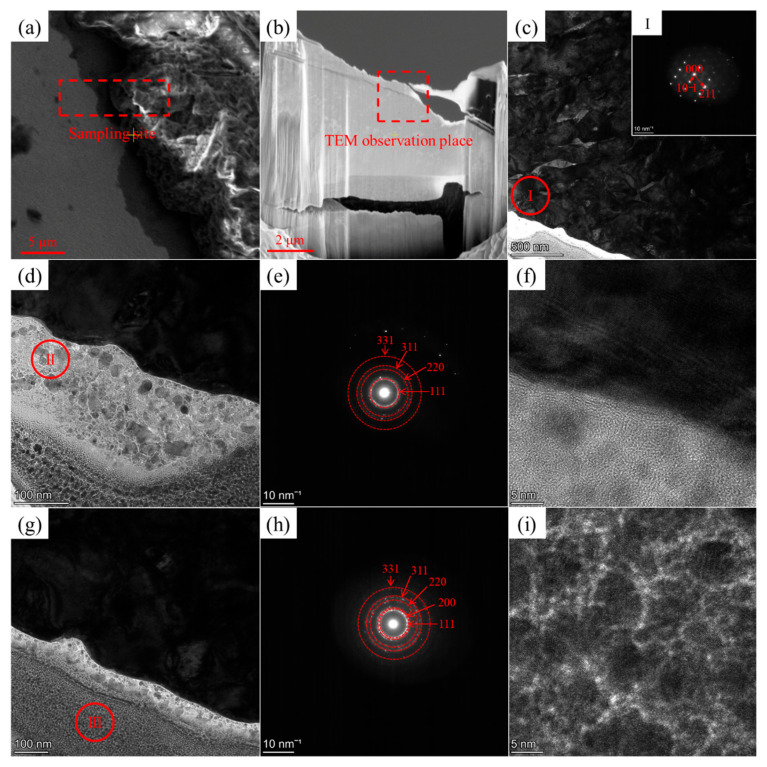
Phase structure analysis of the W and carbon phase interface in the W+(D-Ni) sample: (**a**) TEM sampling site; (**b**) TEM sample prepared by FIB; (**c**) bright-field TEM image of the interface—upper side is the W phase, lower side is the carbon phase, with the diffraction pattern of the W phase shown in image I; (**d**) bright-field TEM image of the middle area in the carbon phase side; (**e**) diffraction pattern of area II; (**f**) HRTEM of area II; (**g**) bright-field TEM image of the bottom area in the carbon phase side; (**h**) diffraction pattern at area III; (**i**) HRTEM of area III.

The phase structure at the W and carbon phase interface in the W+(D-Ni) sample is shown in Figure 8. The W phase is still a β-bcc structure. Compared to that of the W+D sample, the grain size of the W phase is refined, and the dendrite width is approximately at the sub-micron scale, as shown in Figure 8c. There are two different morphologies in the carbon phase at the interface, as shown in Figure 8d,e. In the middle area of the carbon phase, grains are embedded in the amorphous phase, with grain sizes about tens of nanometers, as shown in Figure 8d. The nanocrystalline rings can be found in the diffraction pattern of area II (Figure 8e). In the bottom area of the carbon phase, more and smaller nanocrystals of the cubic diamond phase are observed, confirmed by the diffraction pattern and HRTEM in area III, as shown in Figure 8g–i.

**Figure 9:**
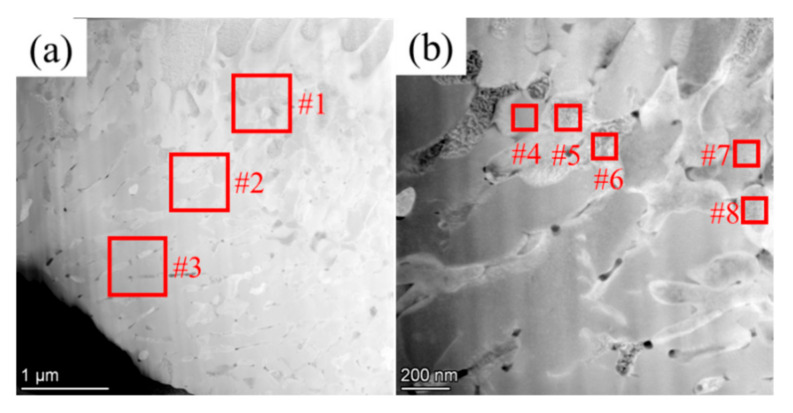
(**a**) and (**b**) are Dark-field TEM images of the W phase at the interface in the W+(D-Ni) sample. Composition of Zone #1–#8 is shown in Table 3 and Table 4.

Figure 9 and Table 3 and Table 4 show the dark-field TEM images of the W phase at the interface in the W+(D-Ni) sample. The dendritic morphology of the W phase can be clearly observed. The C element diffuses from 11.06 at% (Zone #3) to 6.35 at% (Zone #1) in the W phase, while the Ni element (Zone #1 to #3) is about 3 at%, as shown in Figure 9a and Table 3. There exist obvious compositional differences between the W phase dendrites, as shown in Figure 9b and Table 4. For example, the C element is 3.2 at% on average in the W phase of Zone #5, #6, and #8, while it is 8.39 at% on average in Zone #4 and #7.

**Figure 10:**
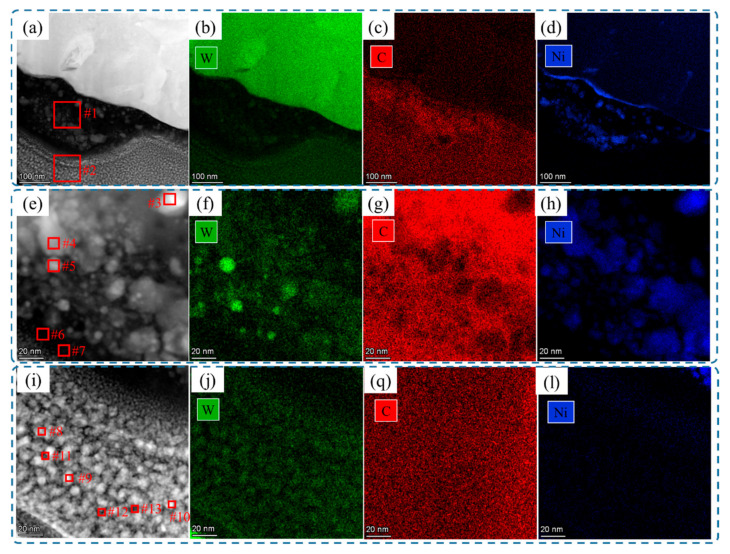
Dark-field TEM and EDX images of the carbon phase at the interface in the W+(D-Ni) sample: (**a**) Dark-field TEM image of the interface; (**b**–**d**) EDX mappings of figure (**a**); (**e**) Dark-field TEM image of the middle area in the carbon phase; (**f**–**h**) EDX mappings of figure (**e**); (**i**) Dark-field TEM image of the bottom area in the carbon phase; (**j**–**l**) EDX mappings of figure (**i**). Composition of Zone #1–#13 is shown in Table 5 and Table 6.

In the W+(D-Ni) sample, there exist obvious compositional differences between the middle area and the bottom area of the carbon phase at the interface. The middle area is more enriched in Ni element than the bottom area, as shown in Figure 10a–d and Table 5. For example, the Ni element concentration in Zone #1 in the middle area is 16.09 at%, but only 1.34 at% in Zone #2 of the bottom area. In the middle area, nanocrystals with sizes from a few nanometers to several hundred nanometers are embedded in the amorphous phase, which is rich in Ni element, as shown in Figure 10e–h and Table 6. For example, the Ni element is 29.87 at% on average in the nanocrystals (Zone #3, #4, and #5), while only 1.3 at% on average in the amorphous phase (Zone #6 and #7). In the bottom area, the volume fraction of nanocrystals is larger, but the size is approximately tens of nanometers, as shown in Figure 10i–l and Table 6. Compared to the middle area, due to the lower Ni element content of the bottom area, the Ni element content of nanocrystals is 2.21 at% on average in Zones #8, #9, and #10, which is also slightly higher than that of the amorphous phase (1.06 at% on average) in Zones #11, #12, and #13.

### 3.4. Bond Composition of Raman Spectrum

**Figure 11:**
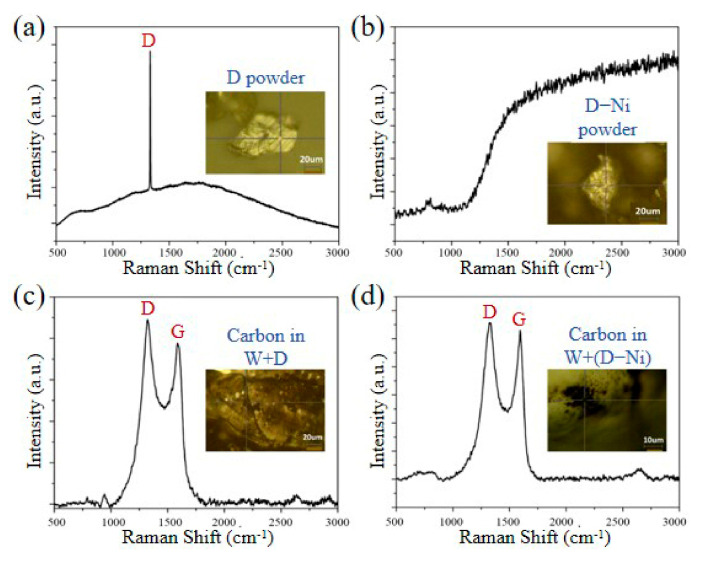
Raman spectra: (**a**) D powder, (**b**) D-Ni powder, (**c**) carbon phase in W+D sample, (**d**) carbon phase in W+(D-Ni) sample.

The Raman spectra are applied to analyze the C bond composition of the D and D-Ni powders, W+D, and W+(D-Ni) samples, as shown in Figure 11. In the D powder, the D peak (1330 cm^−1^) of the sp^3^ bond is the only characteristic peak, indicating the cubic structure of the diamond powder, as shown in Figure 11a. In the D-Ni powder, there are no characteristic peaks due to the Ni coating on the diamond, as shown in Figure 11b. Compared to the Raman spectra of the D powder, the G peak (1600 cm^−1^) of the sp^2^ bond exists in the Raman spectra of carbon phases near the interface in the W+D and W+(D-Ni) samples, as shown in Figure 11c,d. The D and G peaks are both broadened, and the sp^2^ bond ratio is slightly higher than that of the sp^3^ bond. According to relevant research [26], the carbon phases are amorphous at the interface in the W+D and W+(D-Ni) samples, and they are the diamond-like carbon (DLC) phase (ta-C structure), which is also consistent with the XRD and TEM test results (Figure 4, Figure 5, Figure 6 and Figure 8).

## 4. Discussion

### 4.1. Melt Flow and Element Diffusion Behavior

(1) The interface of W+D sample

**Figure 12:**
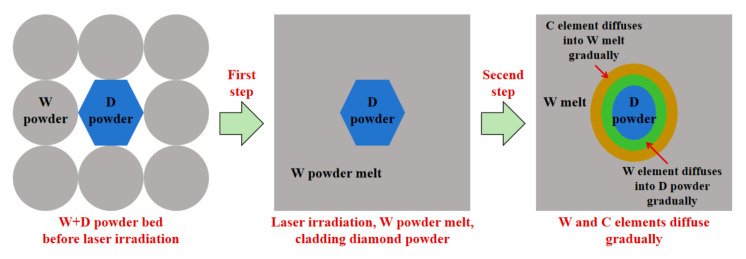
Melting and diffusion process at the interface between W and D powders by laser melting of W+D sample.

At the interface of the W+D sample during laser irradiation, diamond undergoes graphitization and then vaporization under atmospheric pressure [29]. Diamond powder is generally considered to maintain a solid state during the SLM process [18,19,20,21,22,23,24]. Therefore, when exposed to laser irradiation, the W powder melts and envelopes the D powder, as shown in Figure 12. Due to the limited melting time, the C element diffuses into the tungsten melt, the W element diffuses into the diamond powder, and there is no mixture of W and C melts. During the short melting and diffusion time of the laser process, W and C elements cannot be mixed and diffused sufficiently, so a gradient distribution of W and C elements is formed at the interface [30,31,32], as shown in Figure 7 and Table 2 (Zone #1 to #4). In addition, due to the higher cooling rate of SLM processing, the W phase is a supersaturated solid solution of the C element, and the C element content is much greater than that in the equilibrium solidification state (0.7 at%) [33,34,35].

(2) The interface of W+(D-Ni) sample

**Figure 13:**
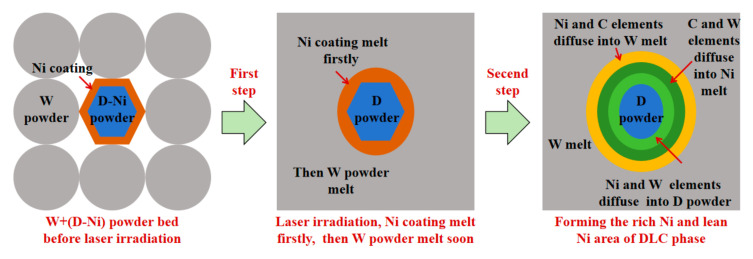
Melting and diffusion process at the interface between W and D-Ni powders during laser melting of W+(D-Ni) sample.

As shown in Figure 12, at the interface of the W+(D-Ni) sample during laser irradiation, the Ni coating melts first, followed shortly by the W powder, while the D powder remains in a solid state, as shown in Figure 13. In the W melt at the interface of the W+(D-Ni) sample, both Ni and W elements are in a molten state, and the mixing and diffusion of Ni and W elements are relatively sufficient. Therefore, there is no obvious element segregation of the Ni element in the W phase at the interface, as shown in Figure 9a and Table 3 (Zone #1 to #3). Due to the solid state of the D powder, there is no mixing of C and W melts, and the C element gradually diffuses into the tungsten melt at the interface, as shown in Figure 9a and Table 3 (Zone #1 to #3). In addition, due to the addition of the Ni element, there is C element segregation between W dendrites in the W+(D-Ni) sample, which is mainly related to the C exclusion reaction during the growth of W dendrites [35], as shown in Figure 9b and Table 4 (Zone #4 to #8).

On the other hand, C and W elements diffuse into the Ni melt at the interface of the D powder, forming a rich Ni area of the DLC phase. Since the diffusion capacity of Ni and W elements in the solid state is significantly weaker than in the molten state, Ni and W elements diffuse into the D powder of the solid state, forming a lean Ni area of the DLC phase, as shown in Figure 10a–d and Table 5 (Zone #1 to #2). The addition of Ni element leads to nanocrystalline precipitation in the DLC phase [28,36,37], with Ni element being enriched in nanocrystals, as shown in Figure 10e–l and Table 6 (Zone #3 to #13). In the rich Ni area of the DLC phase, where element diffusion capacity is stronger in the molten state, Ni elements can achieve a higher degree of enrichment during the solidification segregation process, and the nanoparticles are also larger in size. In the lean Ni area of the DLC phase, compared to the molten state, the element diffusion capacity of the solid state is weaker, Ni elements can only be enriched in the range of tens of nanometers, and the nanoparticles are also smaller.

### 4.2. Microstructure Evolution and Amorphization Mechanism

(1) The effect of Ni coating on phase precipitation

According to the XRD results, there are mainly W, W_2_C, WC_1−x,_ and carbon phases in the W+D and W+(D-Ni) samples, as shown in Figure 4 and Figure 5. W_2_C and WC_1−x_ are carbide phases formed during SLM processing [33,34]. In the W+(D-Ni) sample, the W phase is the dominant phase and the W_2_C is the secondary phase. In the W+D sample, the volume fraction of the W phase decreases, and the W_2_C phase increases. The main reason for less W_2_C phase precipitation in the W+(D-Ni) sample is that the Ni coating of the D-Ni powder delays and reduces the diffusion between the W matrix phase and the C element.

(2) DLC phase formation and nanocrystalline precipitation

During SLM processing of the W+D sample, the cooling rate of the SLM molten pool is large, which leads to the DLC phase forming at the interface between the W phase and diamond powder. Laser deposition is one of the common methods used for preparing DLC coatings [27,35,36]. The bond energy between W, Ni, and C is largely different, and the heat of mixing values [37] of W-C, Ni-C, and W-Ni are −60 kJ/mol, −39 kJ/mol, and −3 kJ/mol. These bond energy differences can lead to Ni element segregation during SLM processing of the W+(D-Ni) sample, resulting in the precipitation of nanocrystals in the DLC phase [28,38,39,40].

In addition, the microstructures of the rich Ni and lean Ni areas are also different, as shown in Figure 10. In the rich Ni middle area of the DLC phase, due to the stronger element diffusion capacity in the molten state, there is obvious Ni element segregation between nanocrystals and amorphous matrix phases. Nanocrystals can grow to several hundred nanometers. In the lean Ni bottom area of the DLC phase, the Ni and W element diffusion capacity within the solid D powder is limited. During SLM processing, the nanocrystalline size is only about tens of nanometers.

(3) The effect of Ni coating on W phase at the interface

W has a high ductile-to-brittle transition temperature (DBTT) and lacks room-temperature ductility, which leads to numerous cracks in the W+D sample [41,42]. Compared with the W+D sample, the Ni element can make the W phase grains finer at the interface of the W+(D-Ni) sample, and the W dendrite width is at the submicron scale [43,44,45], as shown in Figure 8 and Figure 9. Furthermore, the Ni element can also reduce the ductile-to-brittle transition temperature of the W phase, cause solid solution softening in the W phase, promote screw dislocation slipping, and improve the fracture toughness of the tungsten phase [43,44,45]. Therefore, the crack defects in the W+(D-Ni) specimen are obviously less than those in the W+D specimen.

In addition, due to the W phase grain refinement effect of the Ni element, spinodal decomposition occurs during W dendrite growth under rapid solidification conditions (such as magnetron sputtering and laser processing). The supersaturated solid solution of the W phase will expel C elements, forming W dendrites with rich C and lean C regions [33,34,38]. For example, the C content is high (8.39 at% on average) in W dendrites (Figure 9b zones #4 and #7), but low (3.2 at% on average) in adjacent W dendrites (Figure 9b zones #5, #6 and #8). This expulsion of C can further promote the refinement of W-phase grains [38].

## 5. Conclusions

(1) At the interface of the W+D sample, the W powder melts while the D powder remains in the solid state during the L-PBF processing. W and C elements gradually diffuse into each other. Due to the high cooling rate of L-PBF processing, the C phase forms a DLC phase with an amorphous structure, and the W phase becomes a supersaturated solid solution of the C element.
(2) At the interface of the W+(D-Ni) sample, the Ni coating melts first, followed by the W powder melting shortly afterward, while the D powder remains in the solid state during the L-PBF processing. C and W elements diffuse into the Ni melt, forming a rich Ni area of the DLC phase. The diffusion capacity of Ni and W elements in the solid state is weaker than in the molten state; however, they still diffuse into the solid D powder, forming a lean Ni area of the DLC phase.
(3) In the rich Ni area of the DLC phase, the bond energy differences among W-C, Ni-C, and W-Ni lead to Ni segregation in the melt and the precipitation of nanocrystals (several hundred nanometers) in the DLC phase. Compared with the rich Ni area of the DLC phase, the diffusion capacity of Ni and W elements in the solid D powder is limited, resulting in nanocrystalline sizes of only about tens of nanometers in the lean Ni area of the DLC phase.
(4) During W dendrite growth, the addition of Ni element and the expelling of the C phenomenon leads to W grain refinement at the interface, which reduces both the number and length of cracks in the W+(D-Ni) sample.

## Figures and Tables

**Table 1:** Sizes of the raw powder materials used in this study.

Material	Powder Size	Supplier
W powder	15 µm to 45 µm	TEKNA Advanced Materials Inc., Sherbrooke, QC, Canada
D powder	25 µm to 38 µm	HongXiang Superhard Material Co., Ltd., Shangqiu, China
D-Ni powder	25 µm to 38 µm	HongXiang Superhard Material Co., Ltd., Shangqiu, China

**Table 2:** Composition of marked zones in Figure 7.

Average Composition	C (at%)	W (at%)
Zone #1	8.45	91.55
Zone #2	11.62	88.38
Zone #3	97.68	2.32
Zone #4	98.46	1.54

**Table 3:** Composition of marked zones in Figure 9a.

Average Composition	W (at%)	C (at%)	Ni (at%)
Zone #1	90.93	6.35	2.72
Zone #2	89.11	7.82	3.07
Zone #3	86.15	11.06	2.79

**Table 4:** Composition of marked zones in Figure 9b.

Average Composition	W (at%)	C (at%)	Ni (at%)
C rich zone (W phase #4 and #7)	89.71	8.39	1.9
C lean zone (W phase #5, #6, and #8)	93.83	3.2	2.97

**Table 5:** Element ratio of the marked zones in Figure 10a.

Average Composition	C (at%)	W (at%)	Ni (at%)
Zone #1	81.30	2.61	16.09
Zone #2	94.09	4.57	1.34

**Table 6:** Element ratio of the marked zones in Figure 10e–i.

Average Composition	C (at%)	W (at%)	Ni (at%)
Ni rich in nanocrystals (middle area #3, #4, and #5)	62.58	7.55	29.87
Ni lean in amorphous phase (middle area #6 and #7)	97.64	1.06	1.3
Ni rich in nanocrystals (bottom area #8, #9, and #10)	92.74	5.05	2.21
Ni lean in amorphous phase (bottom area #11, #12, and #13)	97.48	1.46	1.06

## Data Availability

The original contributions presented in this study are included in the article. Further inquiries can be directed to the corresponding author.
